# Bilateral Lower Extremity Paralysis in a Caucasian Male Presenting to the Emergency Department

**DOI:** 10.1155/2018/5740509

**Published:** 2018-05-15

**Authors:** Yicheng K. Bao, Vishwanath C. Ganesan, Richard Rapp, Shunzhong S. Bao

**Affiliations:** ^1^University of Missouri-Kansas City School of Medicine, USA; ^2^Little Rock Diagnostic Clinic, USA

## Abstract

Reported is a case of a 39-year-old Caucasian man who presented to the emergency department with sudden onset bilateral lower extremity paralysis after consuming a large amount of carbohydrates and alcohol. A CT, MRI, and lumbar puncture were performed with negative results; lab results showed hyperthyroidism and hypokalemia. The patient was diagnosed with thyrotoxic periodic paralysis. In a patient presenting with sudden onset paralysis and hypokalemia, the emergency physician should include thyrotoxic periodic paralysis in the differential diagnosis and focus on treating and working up the hypokalemia instead of the paralysis.

## 1. Case

A 39-year-old Caucasian male presented to the emergency department with sudden onset bilateral lower extremity paralysis, with normal sensation. He denied any abnormalities in his upper extremities, speech, or vision, and he also denied headache, fever, nausea, or vomiting. The patient consumed large carbohydrate meals with alcohol the night before at a Halloween party, and the patient reported stress from losing his job and separating from his wife. In further questioning, he reported anxiety, tremors, with weight loss of 9.1 kg in past 6 months. He did not take any medications and denied illicit drug use.

Physical examination shows an anxious man with BMI 29.27 kg/m^2^, blood pressure 153/79 mmHg, temperature of 36.8°C, and respiratory rate 18/min. The patient had normal S1 and S2 with irregular beats, normal cranial nerve function, and normal upper extremities. Muscle strength of bilateral lower extremity is 3/5 with normal sensation. Tremors are positive in the upper extremity. Initial BMP revealed K 1.7 mEq/L (normal 3.5–5.1 mEq/L), Na 140 mEq (136–146 mEq), Cl 108.7 (98–107 mEq), CO_2_ 22 mEQ/L (22–31 mEq/L), glucose 147 mg/L (75–110 mg/L), Ca 9.8 mg (normal 8.4–10.2 mg/L), Creatinine 0.65 mg/dl (0.75–1.25 mg/dl), Magnesium 1.66 (1.3–2.1 mEq/L), total CK 330 (30–200 IU/L).

Initial EKG revealed ventricular heart rate of 66 BPM, atrial rate of 234 BPM, atrial flutter with variable A-V premature ventricular beats and aberrantly conducted complexes, prolonged QT of 474 ms, QTc 496 ms. Brain CT, MRI, and lumbar puncture were obtained and found to be normal.

Thyroid function was obtained: TSH < 0.003 (0.35–4.94 uIU/ml), T3 uptake 37 (15–50%), total T4 (4.87–11.72 ug/dl), T7 (Ft4) 4.29 (1.65–4.07 ug/dl), TSI 465 (<140%). Thyroid ultrasound revealed normal thyroid size, but with hypervascular heterogeneous parenchyma, suggesting hyperthyroidism. No nodules were identified.

The patient was treated with intravenous potassium and metoprolol initially, then propranolol and weakness gradually recovered in 12 hours. For the patient's hyperthyroidism, he was treated with methimazole and then had total thyroidectomy.

## 2. Discussion

Thyrotoxic periodic paralysis is rare in Caucasians and typically presents in middle aged Asian men with recurring episodes of sudden onset bilateral lower extremity weakness that can range from weakness to complete paralysis [1]. The upper extremities and muscles controlled by cranial nerves are generally not affected. Decreased muscle tone with hyporeflexia or areflexia is typical [2]. Sensory function is typically normal, and there is no bowel or bladder control dysfunction. Respiratory muscles are usually not affected, but in severe cases they can be paralyzed, causing respiratory failure [3]. Hypokalemia can also cause severe ventricular arrhythmia, A-V block, and ventricular fibrillation [4]. Common triggers include high carbohydrate diet and alcohol, with episodes most often occurring in the morning [4]. In clinic reports, excessive exercise, trauma, emotional stress, acute upper respiratory infection, exposure to cold, use of drugs such as corticosteroids, epinephrine, and NSAIDs are common precursors to thyrotoxic periodic paralysis [5, 6].

The patient consumed large amounts of carbohydrates with alcohol the night before, which is a common precursor of thyrotoxic periodic paralysis. If a patient presents with sudden onset weakness and with severe hypokalemia, thyroid function should be obtained even when there are no obvious symptoms of hyperthyroidism.

### 2.1. Differential Diagnosis

The differential diagnosis should focus on hypokalemia and the cause of hypokalemia. Thyrotoxic periodic paralysis is not a condition of net potassium deficiency; rather it is a condition of transcellular shift of potassium [4, 7]. In this condition, the physician should exercise caution not to overzealously replace potassium, because this can cause hyperkalemia. Other causes for transcellular shift are drugs such as tocolytics, theophylline toxicity, chloroquine toxicity, insulin overdose, beta agonist overdose, and familial hypokalemic periodic paralysis. In this case, the emergency physician should also consider refeeding syndrome in the differential diagnosis. In refeeding syndrome, high intake of carbohydrates and alcohol could cause increases in insulin secretion, which leads to glycogen, fat, and protein synthesis [8]. These processes could consume cofactors such as thiamine and disturb electrolytes, resulting in intracellular potassium shift (hypokalemia) and thiamine deficiency, which could manifest themselves as paralysis, cardiac arrhythmias, and acute Wernicke's encephalopathy [8].

In our case, the potassium was properly replaced and monitored, but he was excessively worked up with a head CT, head and spine MRI, lumbar puncture, and electromyogram. In a presentation of hypokalemia and paralysis, the differential diagnosis should focus on hypokalemia; the extensive workup on paralysis should be held until potassium levels recover.

### 2.2. Pathophysiology

The Na-K ATPase channel has been implicated in TPP, because thyroid hormone increases the Na-K ATPase and shift K into the cells [7]. High carbohydrate meals and alcohol increase insulin levels, which stimulates Na-K ATPase activity [4, 7]. Catecholamines can also increase Na-K ATPase activity in skeletal muscles; therefore stress can also facilitate the attack [4]. Our case has typical triggers, such as stress from job loss and separation from a spouse, as well as a large high carbohydrate meal and high alcohol intake the night before the episode.

Androgens have been suggested to increase the expression of the Na-K ATPase and explain the male to female prevalence to have been reported to be from 20 to 44 : 1 [5, 9], although the prevalence of hyperthyroidism of male to female is 1 : 10. It also suggested that the high level of testosterone and catecholamines in the morning may be responsible for the higher rate of TPP in the morning [4]. Our patient had attack early in the morning which is typical.

## 3. Treatment

Acute treatment requires correction of hypokalemia and control of hyperthyroidism; intravenous potassium with normal saline is recommended. In correction for hypokalemia, attention should be paid to avoid overcorrection with rebound hyperkalemia. Close monitoring is very important, especially when cardiac arrhythmia is present.

Nonspecific beta blockers like propranolol are very effective and should be initiated immediately. Some specialists recommend just using nonspecific beta blocker without potassium supplement to avoid potassium rebound. In this case, high dose (3-4 mg/kg orally) has been reported to be used successfully.

Thyroid function control has been achieved with conventional treatment with medication, surgery, or radioactive iodine. Patients need to be consulted to avoid a high carbohydrate diet, high alcohol intake, or excessive physical exertion even after hyperthyroidism has been controlled.

## 4. Conclusion

TPP is most commonly occurs in middle aged Asian men, but we presented a case of Caucasian man. His hyperthyroidism was not previously diagnosed, and this episode was preceded by high carbohydrate and alcohol. In the ED, typical presentations of paralysis and profound hypokalemia should prompt the physician to check thyroid function. The differential diagnosis should be focused on hypokalemia instead of paralysis.
